# XinJiaCongRongTuSiZiWan protects triptolide-induced rats from oxidative stress injury via mitophagy mediated PINK1/Parkin signaling pathway

**DOI:** 10.1590/acb391424

**Published:** 2024-03-15

**Authors:** Yan Jin, Deng Di-si, Wu Ke-ming

**Affiliations:** 1 Shaanxi University of Chinese Medicine – Xianyang, China.; 2 Shaanxi Key Laboratory of Chinese Medicine – Research on Physical Constitution and Diseases – Xianyang, China.; 3 Hospital of Chengdu University of Traditional Chinese Medicine – Department of Gynecology – Chengdu, China.

**Keywords:** Medicine, Chinese Traditional, Reproductive Medicine, Oxidative Stress, Mitophagy

## Abstract

**Purpose:**

XinJiaCongRongTuSiZiWan (XJCRTSZW) is a traditional Chinese medicine compound for invigorating the kidney, nourishing blood, and promoting blood circulation. This study aimed to explore the effect of XJCRTSZW on triptolide (TP)-induced oxidative stress injury.

**Methods:**

Adult female Sprague-Dawley rats and human ovarian granulosa cell lines were treated with TP and XJCRTSZW. Hematoxylin and eosin staining, enzyme-linked immunosorbent assay, flow cytometry, CCK-8, JC-1 staining, transmission electron microscopy, reverse transcription-quantitative polymerase chain reaction, and Western blotting were performed in this study.

**Results:**

XJCRTSZW treatment observably ameliorated the TP-induced pathological symptoms. Furthermore, XJCRTSZW treatment observably enhanced the TP-induced reduction of estradiol, anti-Mullerian hormone, progesterone, superoxide dismutase, ATP content, mitochondrial membrane potential, p62, and Hsp60 mRNA, and protein levels *in vivo* and *in vitro* (*p* < 0.05). However, TP-induced elevation of follicle stimulating hormone and luteinizing hormone concentrations, malondialdehyde levels, reactive oxygen species levels, apoptosis rate, mitophagy, and the mRNA and protein expressions of LC3-II/LC3-I, PTEN-induced kinase 1 (PINK1), and Parkin were decreased (*p* < 0.05). In addition, XJCRTSZW treatment markedly increased cell viability *in vitro* (*p* < 0.05).

**Conclusions:**

XJCRTSZW protects TP-induced rats from oxidative stress injury via the mitophagy-mediated PINK1/Parkin pathway.

## Introduction

Triptolide (TP), a diterpene trioxide, is a major component extracted from the Chinese herb *Tripterygium wilfordii* Hook F^1^, which has been widely used for the treatment of autoimmune, inflammatory diseases, and a variety of tumors^2^^–^ 4. However, plenty of studies have exhibited severe reproductive system toxicity in TP-treated animals and patients^5^^,^ 6. TP can significantly induce ovarian damage, resulting in follicular atresia, a decrease in the number of corpus luteum and in the gonad index, and the destruction of ovarian microstructure^7^^,^ 8. Oxidative stress is one of the core molecular mechanisms involved in the toxicity of TP, and has been demonstrated to be tightly associated with follicular atresia and ovulation disorders, including premature ovarian failure (POF) and polycystic ovary syndrome (PCOS)^9^^,^ 10. Therefore, oxidative stress may be a plausible target for the prevention or treatment of TP-induced reproductive toxicity, and pharmacologic oxidative stress modulators may be promising drug candidates.

Except for apoptosis, a major hallmark of TP-induced reproductive toxicity, other forms of programmed cell death (PCD), such as autophagy, can be also activated primarily in granulosa cells (GCs)^9^. Autophagy is a highly conserved and closely modulated process that transports cellular substrates to lysosomes for bulk degradation, including macroautophagy, mitophagy, and chaperone-mediated autophagy. Among them, mitophagy degrades the damaged mitochondria, which acts as an effector of PCD and promotes cell death procedures.

Moreover, previous studies have revealed that the oxidative stress-induced mitochondrial permeability transition induces the depolarization of mitochondrial membrane potential, which leads to the accumulation of PTEN-induced kinase 1 (PINK1) on the outer mitochondrial membrane. Subsequently, PINK1 recruits Parkin, an E3 ubiquitin ligase, to initiate autophagic degradation of damaged mitochondria. Thus, mitophagy may play an important role in the TP-induced oxidative stress response.

XinJiaCongRongTuSiZiWan (XJCRTSZW) is a traditional Chinese medicine compound for invigorating the kidney, nourishing blood, and promoting blood circulation. It was created by Professor Keming Wu according to many years of clinical experience. The prescription contains *Cistanches Herba* (Roucongrong), *Cuscutae Semen* (Tusizi), *Rubi Fructus* (Fupenzi), *Taxilli Herba* (Sangjisheng), *Rehmanniae Radix Praeparata* (Shudihuang), *Angelicae Sinensis Radix* (Danggui), *Epimedii Folium* (Yinyanghuo), *Spatholobi Caulis* (Jixueteng), *Cyperi Rhizoma* (Xiangfu), *Leonuri Fructus* (Chongweizi), *Lycii Fructus* (Gouqizi), *Lycopi Herba* (Zelan), *Dioscoreae Rhizoma* (Shanyao), and *Corni* *Fructus*(Shanzhuyu). *Cistanches Herba*, *Cuscutae Semen*, and *Rubi Fructus* are used as emperor medicines to invigorate the kidney and essence. *Epimedii Folium*, *Corni Fructus*, and *Rehmanniae Radix Praeparata* act as minister medicines to strengthen the power of the emperor medicines to nourish the kidney. Among them, *Epimedii Folium* warms the kidney and yang, and *Corni Fructus* and *Rehmanniae Radix Praeparata* nourish the essence and blood of the liver and kidney. The rest of the medicines are all adjuvants. Among them, *Angelicae Sinensis Radix*, *Cuscutae Semen*, *Lycii* *Fructus*, and *Lycopi Herba* nourish blood and promote blood circulation; *Taxilli Herba* nourishes the liver and kidneys, strengthens muscles and bones; *Dioscoreae Rhizoma* nourishes qi and invigorates the spleen; and *Cyperi Rhizoma* is the key to regulating menstruation in gynecology, which can regulate the qi and activate the blood, and make all the medicines tonic without stagnation.

Our group has discovered an effective treatment of XJCRTSZW on POF and PCOS^11^^,^ 12. Furthermore, our previous results have also shown that XJCRTSZW reduces the TP-induced enhancement of apoptosis and macroautophagy. However, the role of XJCRTSZW in TP-induced oxidative stress mediated by mitophagy is still not fully understood.

Therefore, in this study, we investigated the role of XJCRTSZW on the TP-induced oxidative stress mediated by mitophagy both *in vivo* and *in vitro*, and the mechanism of mitophagy mediated by the PINK1/Parkin signaling pathway in this process. The results of this study will provide new insights and methods for the treatment of reproductive toxicity, even for POF and PCOS.

## Methods

### Animal

Adult female Sprague-Dawley rats (7-8 weeks, 200–220 g) were bought from Chengdu Dashuo Biological Technology Co., Ltd. Rats were acclimated to standard laboratory conditions for seven days before the experiments. Rats were supplied with a 12 h/12 h light-dark cycle and fed with standard diet and water *ad libitum* at 25 ± 2°C, and 40–60% relative humidity. All the procedures were executed severely according to the Board and Ethics Committee of Chengdu University of Traditional Chinese Medicine.

### Cell culture

Human ovarian granulosa cell lines (cat no. CP-H192) were bought from Procell (Wuhan, China). Cells were cultured in complete medium for human ovarian granulosa cells (CM-H192, Procell) at 37°C with 5% carbon dioxide (CO_2_).

### Cell transfection

Pink1 mimics and Pink1 inhibitor were designed and synthesized by Ribobio (Guangzhou, China). It was performed with the RiboFectTM CP Transfection Kit (C10511-05, Ribobio) following the experimental procedures.

### Preparation of medicated serum

For *in-vitro* experiments, medicated serum was first prepared as follows. Fifteen rats were randomly divided into three groups (*n* = 5): the control, estradiol valerate (EV, western medicine control group), and XJCRTSZW groups. Rats in the EV group were intragastrically administered with 0.105-mg/kg/d EV once a day for four consecutive days, followed by 0.63-mg/kg/d medroxyprogesterone acetate once a day for one day. Rats in the XJCRTSZW group were intragastrically administered with 25.2-g/kg/d XJCRTSZW, while rats in the control group were intragastrically administered with 1-mL/100-g saline twice a day for five consecutive days. Subsequently, blood was obtained from the abdominal aorta after the rats were intraperitoneally anesthetized with sodium pentobarbital (40 mg/kg). Serum was isolated and inactivated the complement for *in-vitro* experiments.

### Experimental groups and drug administration

For *in-vivo* experiments, 30 rats were randomly divided into six groups (*n* = 5): the control, TP, TP + EV, TP + XJCRTSZW-High, TP + XJCRTSZW-Middle, and TP + XJCRTSZW-Low groups. Rats in the TP, TP + EV, TP + XJCRTSZW-High, TP + XJCRTSZW-Middle and TP + XJCRTSZW-Low groups were intragastrically administered with 400-μg/kg/d TP (dissolved in saline containing 5% DMSO) for a continuous 30 days to induce reproductive toxicity, while rats in the control group were intragastrically administered with an equal amount of saline. Then, rats in the TP + XJCRTSZW-High, TP + XJCRTSZW-Middle, and TP + XJCRTSZW-Low groups were intragastrically administered with 30-, 15-, and 7.5-g/kg/d XJCRTSZW for two weeks, respectively. Rats in the TP + EV group were intragastrically administered with 0.1-mg/kg/d EV once a day for four consecutive days, followed by 0.598-mg/kg/d medroxyprogesterone acetate once a day for one day. A total of 15 days continued with one day after stopping the drug. Rats in the control and TP groups were intragastrically administered with 1 mL/100 g of saline once a day for two weeks.

The total amount of crude drug for each dose of XJCRTSZW is 120 g, which was made by the Department of Pharmacy of Chengdu University of Traditional Chinese Medicine. The methods were consistent with previous reported^12^. The concentrations of XJCRTSZW in the high, middle, and low groups, as well as EV and medroxyprogesterone acetate, were determined using an adult clinical dose of 6 kg per kilogram of body weight and a human to rat conversion ratio of 1:20. After the rats were intraperitoneally anesthetized with sodium pentobarbital (40 mg/kg), blood was taken from the abdominal aorta. Serum was isolated and stored at -80°C for further assays. Ovary tissues and GCs were rapidly removed for subsequent analysis.

For *in-vitro* experiments, human ovarian GC lines were inoculated into six-well plates and divided into eight groups, including control, TP, TP + EV, TP + XJCRTSZW, TP + XJCRTSZW + si-Pink1, TP + XJCRTSZW + ov-Pink1, TP + si-Pink1, and TP + ov-Pink1 groups. Human ovarian GCs in the control group were cultured in complete medium for human ovarian GCs, while cells in the other seven groups were cultured in complete medium for human ovarian GCs, including 100 nM TP for 6 h. Then, cells in the TP group were cultured in complete medium for human ovarian GCs, including 200 μL of control serum obtained from control rats, while cells in the TP + EV group were cultured in complete medium for human ovarian GCs, including 200 μL of EV serum obtained from EV rats. Cells in the TP + XJCRTSZW, TP + XJCRTSZW + si-Pink1, and TP + XJCRTSZW + ov-Pink1 groups were cultured in complete medium for human ovarian granulosa cells, including 200 μL medicated serum obtained from rats in the XJCRTSZW group. Subsequently, cells in the TP + XJCRTSZW + si-Pink1 and TP + XJCRTSZW + ov-Pink1 groups were cultured in complete medium for human ovarian GCs, including 20-nM Pink1 inhibitor and 2-μg Pink1 mimics, respectively. Cells in the TP + si-Pink1 and TP + ov-Pink1 groups were cultured in complete medium for human ovarian GCs, including 200-μL control serum obtained from control rats, as well as 20-nM Pink1 inhibitor and 2-μg Pink1 mimics, respectively. Cells were maintained at 37°C with 5% CO_2_ for an additional duration of 48 h.

### Cell counting kit-8 assay

Human ovarian GCs were cultured in 96-well plates with an inoculation density of 1 × 10^5^/well and maintained for 24 h at 37°C in 5% CO_2_. Then, the Cell Count Kit-8 (Dojindo Laboratories, Kumamoto, Japan) was utilized to determine the proliferation of cells based on the manufacturer’s specifications. The absorbance was recorded at 450 nm using a microplate reader (Thermo Fisher Scientific, Waltham, MA, United States of America).

### Histological assays and follicle count

The ovarian tissues were separated, fixed, decalcified, embedded, and cut into sections. Five-μm sections were stained with hematoxylin and eosin (H&E). Pictures were obtained under a microscope (DMI1, LEICA, Germany). Then, the number of primary follicles, secondary follicles, and atretic follicles was measured.

### Enzyme-linked immunosorbent assay

The levels of estradiol (E2), anti-Mullerian hormone (AMH), follicle stimulating hormone (FSH), luteinizing hormone (LH), progesterone (P), and ATP were detected using the Estradiol Enzyme-linked immunosorbent assay (ELISA) Kit (PE223, Beyotime, Shanghai, China), Rat AMH ELISA Kit (YB-AMH-Ra, Ybscience, Shanghai, China), Rat FSH ELISA Kit (XY-FSH-Ra, Ybscience), Rat LH ELISA Kit (XY-LH-Ra, Ybscience), Rat Progesterone, PROG ELISA Kit (KB3091, Kemin Biology, Shanghai, China), and Rat ATP ELISA Kit (Qingqi Biology, Shanghai, China), according to the manufacturer’s protocol. The absorbance of wells was determined with a microplate reader (Thermo Fisher Scientific) at 450-nm wavelength to analyze the sample concentrations.

### Biochemical detection

The levels of malondialdehyde (MDA) and superoxide dismutase (SOD) were detected using the Lipid Peroxidation MDA Assay Kit (S0131M, Beyotime) and the Total Superoxide Dismutase Assay Kit with NBT (S0109, Beyotime), according to the manufacturer’s protocol. The absorbance of wells was determined with a microplate reader (Thermo Fisher Scientific) at 532-nm (MDA) and 560-nm (SOD) wavelengths to analyze the sample concentrations.

### Flow cytometric assay

Apoptosis of GCs was evaluated using flow cytometric assay. In brief, GCs were collected and stained with Annexin V-APC and PI (Sigma Aldrich, St. Louis, MO, United States of America) at room temperature for 20 min in the dark. The fluorescence of the cells was measured by flow cytometry (BD FACSVerse, Waltham, MA, United States of America).

The level of reactive oxygen species (ROS) was detected using the Reactive Oxygen Species Assay Kit (S0033, Beyotime, Shanghai, China) according to the operating manual. The fluorescence of the cells was measured by flow cytometry (BD FACSVerse).

### JC-1 staining

GCs cultured on coverslips were maintained with 0.1-μM JC-1 (Molecular Probes) at 37°C for 20 min. Fluorescence images were obtained by a laser-scanning confocal microscope (Zeiss LSM 710 META) with an excitation at 525 and 490 nm, respectively. CCCP (50 mM) was used as a positive control and treated for 20 min before JC-1 staining. Changes in mitochondrial membrane potential (MMP) were analyzed by the ratio of aggregated JC-1 (525 nm, red fluorescence) to monomeric JC-1 (490 nm, green fluorescence).

### Transmission electron microscopy

Ovary tissues and GCs were fixed in 3% glutaraldehyde and 1% osmium tetroxide and cut on an ultramicrotome. Then, sections were successively stained with 1% uranyl acetate and 0.5% lead citrate. The results were observed using a JEM-1400PLUS transmission electron microscope.

### Reverse transcription-quantitative polymerase chain reaction

Total RNA was isolated using TRIzol reagent (TaKaRa Biotechnology Co., Ltd., Dalian, China), and reverse transcription (RT) was conducted by the Bio-Rad ScripTM cDNA Synthesis Kit (Bio-Rad Laboratories, Inc., Hercules, CA, United States of America), according to the manufacturer’s instructions. The resulting RT products were stored at -80°C until analysis. RT-quantitative polymerase chain reaction (RT-qPCR) was performed in a 20-μL mixture containing 2 μL of the cDNA templates, 10-μL 2x SYBR Master mix (MedChemexpress, Princeton, NJ, United States of America), 0.4 μL of the 10-μM forward and reverse primers and 7.2-μL ddH_2_O, using the Bio-Rad CFX Manager software (Bio-Rad Laboratories, Inc.). The RT-qPCR conditions were as follows: 5 min at 95°C, followed by 40 cycles between 95°C for 15 sec and 60°C for 30 sec, and 72°C for 30 sec. The relative expressions of LC3, PINK1, Parkin, p62, and Hsp60 were calculated using the 2^-ΔΔCT^ method and normalized to the housekeeping gene β-actin. The RT reaction was processed at 42°C for 60 min and at 70°C for 10 min. Gene expression levels were quantified at 95°C for 10 min, followed by 40 cycles at 95°C for 2 sec, 60°C for 20 sec and 70°C for 10 sec. U6 served as the internal control.

### Western blot assay

Protein samples from GCs separated from ovary or human ovarian GCs were extracted using a Total Protein Extraction Kit (BC3711, Solarbio, Beijing, China). Then, the protein concentration was detected by a Protein Assay Kit (Beyotime). Next, protein samples were separated by 10% SDS-PAGE gel and electrically transferred to polyvinylidene difluoride membranes (Millipore, MA, United States of America). After being blocked with 3% bovine serum albumin (BSA) for 1 h at room temperature, the membranes were incubated with the primary antibodies at 4°C overnight. After washing them with TBST for 3 × 5 min, the membranes were incubated with goat-anti-rabbit IgG (H+L)-HRP (1:10,000, ab6721, Abcam, Cambridge, United Kingdom) for 1 h at room temperature. Protein bands were analyzed by an Electrochemiluminescence Kit (WBULS0500; EMD Millipore), and the band intensity was quantified with Image-Pro Plus 6.0 software.

The primary antibodies used were as follows: rabbit anti-LC3II/I (ab128025, 1:1,000, Abcam), rabbit anti-p62 (ab56416, 1:1,000, Abcam), rabbit polyclonal to PINK1 (ab23707, 1:1,000, Abcam), rabbit polyclonal to Parkin (ab15494, 1:1,000, Abcam), rabbit polyclonal to Hsp60 (ab46798, 1:20,000, Abcam), and rabbit anti-β-actin (ab8227, 1:1,000, Abcam).

### Statistical analysis

Data were presented as the means ± standard deviation. Differences among multiple groups were analyzed using one-way analysis of variance and Duncan’s test using the Statistical Package for the Social Sciences 20.0 (SPSS Inc. Chicago, IL, United States of America). The differences were considered statistically non-significant and significant when *p* > 0.05 and *p* < 0.05, respectively.

## Results

### XJCRTSZW ameliorates triptolide-induced in-vivo reproductive toxicity

**Figure 1:**
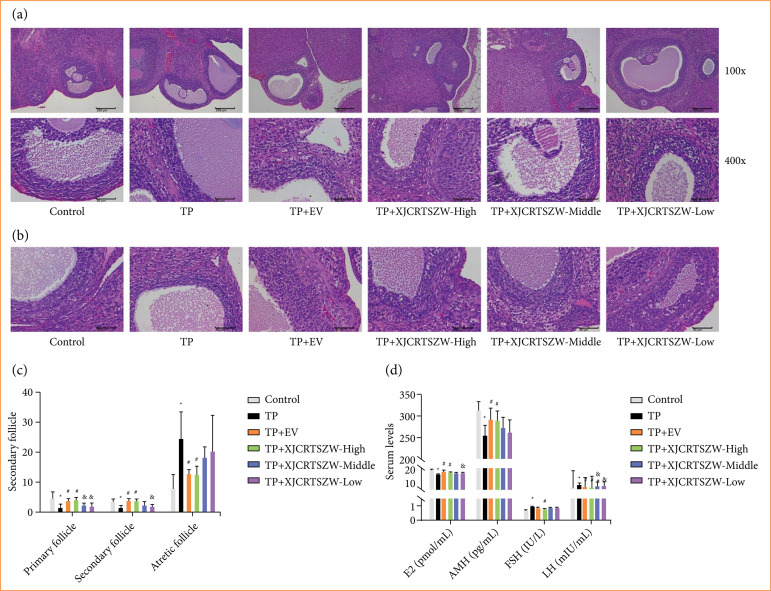
XJCRTSZW ameliorates TP-induced reproductive toxicity. **(a)** Histological analysis of the ovary was determined by hematoxylin and eosin staining. **(b)** The number of primary follicles, secondary follicles, and atretic follicles were measured after ovary was stained with hematoxylin and eosin. **(c)** The serum levels of E2, AMH, FSH, and LH were detected using commercial enzyme-linked immunosorbent assay kits. The means ± standard deviation of five independent samples were shown.

XJCRTSZW: XinJiaCongRongTuSiZiWan; TP: triptolide; EV: estradiol valerate; E2: estradiol; AMH: anti-Mullerian hormone; FSH: follicle stimulating hormone; LH: luteinizing hormone; *p < 0.05 compared to the control group; ^#^ *p* < 0.05 compared to the TP group; ^&^ *p* < 0.05 compared to the TP + XJCRTSZW-High group. Source: Elaborated by the authors.

After rats were treated with TP, pathological analysis revealed that the number of primordial follicles, primary follicles, and secondary follicles in the cortical area decreased, while the number of atretic follicles increased. Moreover, some granular layer follicles were necrotic and shedding, and nuclear constriction and collapse were seen with various degrees of cystic dilatation. However, these pathological symptoms were notably improved after both EV and XJCRTSZW treatment (Fig. 1a).

Furthermore, quantification of follicle numbers showed that both EV and XJCRTSZW treatment significantly enhanced the TP-induced reduction in the number of primary follicles and secondary follicles, while prominently decreasing the TP-induced elevation in the number of atretic follicles (Figs. 1b and 1c). The serum levels of E2 and AMH were observably diminished, whereas FSH and LH levels were markedly increased with TP treatment. However, both EV and XJCRTSZW treatments prominently reversed the decrease of E2 and AMH levels, while only XJCRTSZW treatment significantly antagonized the enhancement of FSH and LH levels (Fig. 1d). Taken together, we demonstrated that XJCRTSZW ameliorates TP-induced reproductive toxicity in the rat model.

### XJCRTSZW inhibits triptolide-induced in-vivo oxidative stress injury

**Figure 2:**
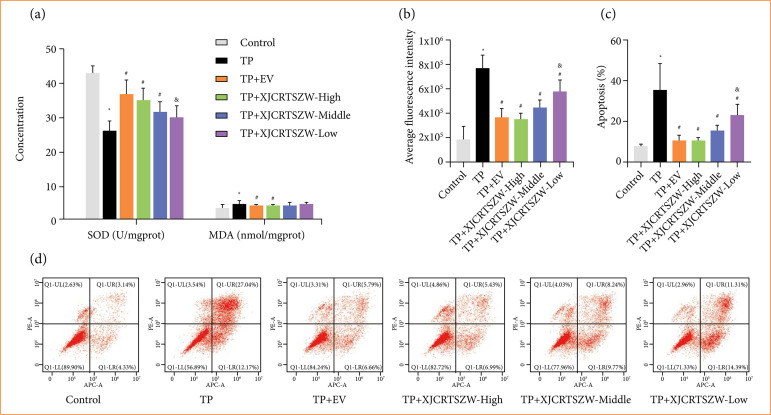
XJCRTSZW inhibits TP-induced *in-vivo* oxidative stress injury and apoptosis. **(a)** Levels of SOD and MDA in granulosa cells were detected using commercial kits. **(b)** Reactive oxygen species levels were determined using the reactive oxygen species assay kit, and the fluorescence of the cells was measured by flow cytometry. (**c**and **d**) The apoptosis rate was analyzed using flow cytometry. The means ± standard deviation of five independent samples were shown.

XJCRTSZW: XinJiaCongRongTuSiZiWan; TP: triptolide; EV: estradiol valerate; SOD: superoxide dismutase; MDA: malondialdehyde; *p < 0.05 compared to the control group; ^#^ *p* < 0.05 compared to the TP group; ^&^ *p* < 0.05 compared to the TP + XJCRTSZW-High group. Source: Elaborated by the authors.

To determine the role of XJCRTSZW in TP-induced oxidative stress injury, we first detected the levels of SOD and MDA. The results showed that all the EV, high, and middle-dose of XJCRTSZW treatment significantly elevated the TP-induced decrease in SOD levels, while only EV and high dose of XJCRTSZW treatment prominently inhibited the TP-induced increase in MDA levels (Fig. 2a). In addition, all the EV, high, middle, and low dose of XJCRTSZW treatments notably antagonized the TP-induced increase in ROS levels (Fig. 2b). Furthermore, TP treatment markedly enhanced the apoptosis rate of GCs, which was observably reversed with all the EV, high, middle, and low dose of XJCRTSZW treatment (Figs. 2c and 2d). Therefore, these results suggested that XJCRTSZW inhibits TP-induced *in-vivo* oxidative stress injury and apoptosis.

### XJCRTSZW promotes mitophagy via the in-vivo PINK1/Parkin signaling pathway

**Figure 3:**
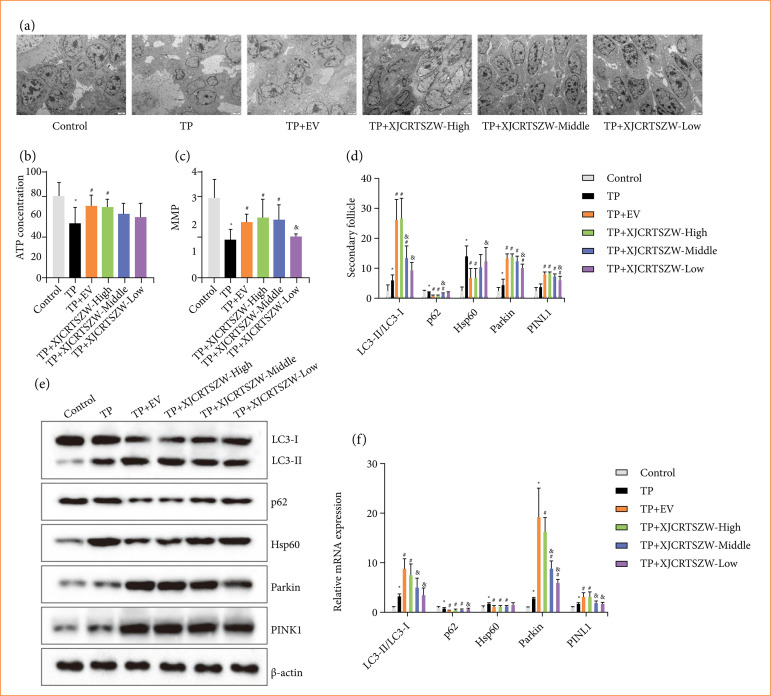
XJCRTSZW promotes mitophagy via the *in-vivo* PINK1/Parkin signaling pathway. **(a)** The mitophagy in granulosa cells was assessed using a transmission electron microscope. **(b)** ATP levels were detected by an enzyme-linked immunosorbent assay. **(c)** MMP was determined by JC-1 staining. (**d** and **e**) The protein levels of LC3-II/LC3-I, p62, Hsp60, Parkin, and PINK1 were evaluated using western blotting. The data were expressed after being normalized to β-actin. **(f)** The mRNA levels of LC3, p62, Hsp60, Parkin, and PINK1 were detected using quantitative reverse transcription polymerase chain reaction. The data were expressed after being normalized to β-actin. The means ± standard deviation of five independent samples were shown.

XJCRTSZW: XinJiaCongRongTuSiZiWan; TP: triptolide; EV: estradiol valerate; PINK1: PTEN-induced kinase 1; MMP: mitochondrial membrane potential; *p < 0.05 compared to the control group, ^#^ *p* < 0.05 compared to the TP group, ^&^ *p* < 0.05 compared to the TP + XJCRTSZW-High group. Source: Elaborated by the authors.

Transmission electron microscopy showed many swollen mitochondria in GCs with broken, dissolved, or even disappeared cristae, and a spot of mitophagy in the TP treatment group, while the EV, high-, middle-, and low-dose XJCRTSZW treatments relieved the degree of mitochondrial swelling and increased the number of mitophagy (Fig. 3a). Besides, the levels of ATP and MMP were observably reduced after TP treatment, which were markedly rescued with EV and high-dose XJCRTSZW treatment (Figs. 3b and 3c). Both EV and high-dose XJCRTSZW treatment prominently increased LC3-II/LC3-I expression, while significantly decreasing p62 and Hsp60 expression both at transcriptional and translational levels compared to those in the TP group (Figs. 3d–3f). Furthermore, the mRNA and protein expression levels of PINK1 and Parkin were notably enhanced with both EV and high-dose XJCRTSZW treatment compared to the TP group. Altogether, we concluded that XJCRTSZW promoted mitophagy via the *in-vivo* PINK1/Parkin signaling pathway.

### XJCRTSZW promotes triptolide-induced cell viability of granulosa cells

**Figure 4:**
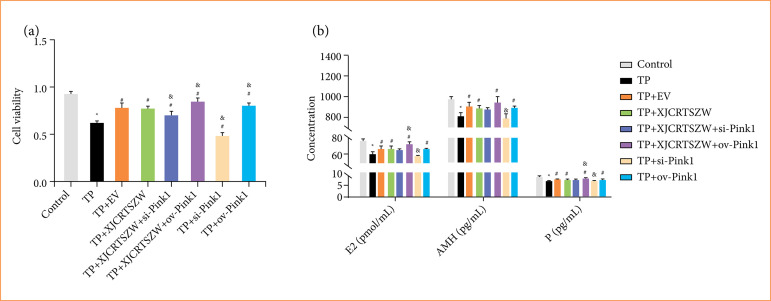
XJCRTSZW promotes the cell viability of granulosa cells induced by TP. **(a)** Cell viability was detected using CCK-8. **(b)** The levels of E2, AMH, and P in the supernatant of cultured human ovarian GCs were determined using commercial enzyme-linked immunosorbent assay kits. The means ± standard deviation of three independent samples were shown.

XJCRTSZW: XinJiaCongRongTuSiZiWan; TP: triptolide; EV: estradiol valerate; PINK1: PTEN-induced kinase 1; E2: estradiol; AMH: anti-Mullerian hormone; P: progesterone; *p < 0.05 compared to the control group, ^#^ *p* < 0.05 compared to the TP group, ^&^ *p* < 0.05 compared to the TP + XJCRTSZW-High group. Source: Elaborated by the authors.

The cell viability of human ovarian GCs was notably declined after TP treatment, while cell viability was prominently rescued after EV or XJCRTSZW treatment. Moreover, further inhibition or overexpression of PINK1 prominently decreased and increased the cell viability based on the enhanced cell viability treated by XJCRTSZW, respectively. Only inhibition or overexpression of PINK1 markedly reduced or elevated the cell viability compared with the TP group, respectively (Fig. 4a). Similarly, EV or XJCRTSZW treatment notably increased the TP-induced reduction of E2, AMH, and P levels in the supernatant of cultured human ovarian GCs. XJCRTSZW treatment combined with inhibition or overexpression of PINK1 further reduced E2, AMH, and P levels (without statistical difference), or significantly increased E2, AMH, and P levels in supernatant of cultured human ovarian GCs. In addition, only inhibition or overexpression of PINK1 significantly decreased or increased E2, AMH, and P levels in the supernatant of cultured human ovarian GCs (Fig. 4b). Thus, we demonstrated that XJCRTSZW promotes the cell viability of GCs induced by TP.

### XJCRTSZW suppresses triptolide-induced in-vitro oxidative stress injury

**Figure 5:**
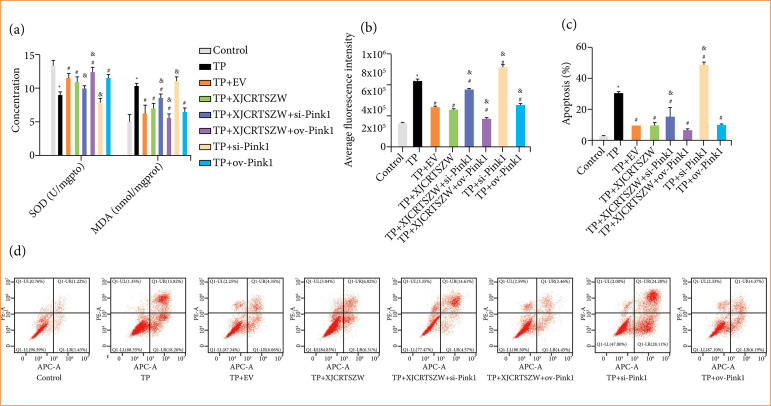
XJCRTSZW suppresses TP-induced *in-vitro* oxidative stress injury. **(a)** The levels of SOD and MDA in granulosa cells were detected using commercial kits. **(b)** Reactive oxygen species levels were determined using the reactive oxygen species assay kit, and the fluorescence of the cells was measured by flow cytometry. **(c and d)** The apoptosis rate was analyzed using flow cytometry. The means ± standard deviation of three independent samples were shown.

XJCRTSZW: XinJiaCongRongTuSiZiWan; TP: triptolide; EV: estradiol valerate; PINK1: PTEN-induced kinase 1; SOD: superoxide dismutase; MDA: malondialdehyde; *p < 0.05 compared to the control group, ^#^ *p* < 0.05 compared to the TP group, ^&^ *p* < 0.05 compared to the TP + XJCRTSZW-High group. Source: Elaborated by the authors.

Like the results shown *in vivo*, EV and XJCRTSZW treatment significantly enhanced the TP-induced diminishment of SOD levels, which was further significantly elevated by XJCRTSZW combined with overexpression of PINK1, but prominently decreased by XJCRTSZW combined with inhibition of PINK1. On the contrary, EV and XJCRTSZW treatment observably diminished the TP-induced elevation of MDA levels, which was further markedly reduced with XJCRTSZW combined with inhibition of PINK1, but prominently increased with XJCRTSZW combined with overexpression of PINK1 (Fig. 5a). Moreover, both the levels of ROS and apoptosis rate of cultured human ovarian GCs were notably increased after TP treatment, which was markedly antagonized by EV and XJCRTSZW treatments. XJCRTSZW treatment combined with inhibition or overexpression of PINK1 prominently enhanced or declined the levels of ROS and apoptosis rate of cultured human ovarian GCs compared to these with XJCRTSZW treatment, respectively (Figs. 5b and 5d). Therefore, these results indicated that XJCRTSZW suppresses TP-induced *in-vitro* oxidative stress injury.

### XJCRTSZW facilitates mitophagy via the PINK1/Parkin signaling pathway in vitro

**Figure 6:**
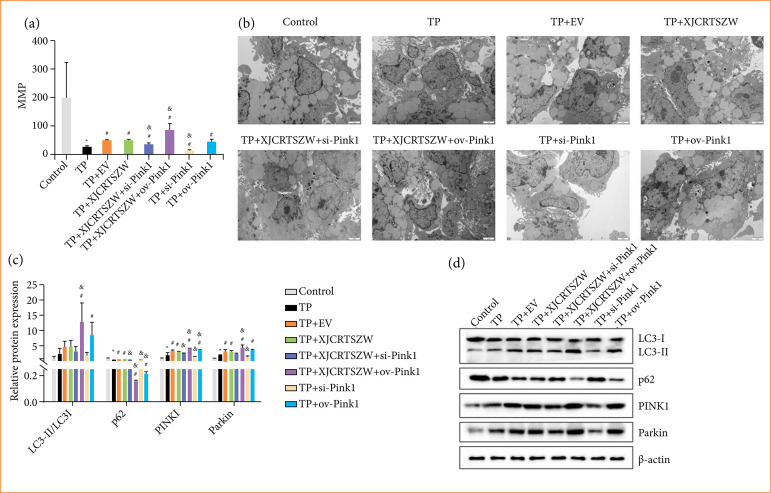
XJCRTSZW facilitates mitophagy via the *in-vitro* PINK1/Parkin signaling pathway. **(a)** MMP was determined by JC-1 staining. **(b)** The mitophagy in granulosa cells was detected using a transmission electron microscope. (**c** and**d**) The protein levels of LC3-II/LC3-I, p62, Parkin, and PINK1 were evaluated using western blotting. The data were expressed after being normalized to β-actin. The means ± standard deviation of three independent samples were shown.

XJCRTSZW: XinJiaCongRongTuSiZiWan; TP: triptolide; EV: estradiol valerate; PINK1: PTEN-induced kinase 1; MMP: mitochondrial membrane potential; *p < 0.05 compared to the control group, ^#^ *p* < 0.05 compared to the TP group, ^&^ *p* < 0.05 compared to the TP + XJCRTSZW-High group. Source: Elaborated by the authors.

The reduction of MMP induced by TP was markedly elevated with EV and XJCRTSZW treatment, and XJCRTSZW treatment combined with inhibition or overexpression of PINK1 prominently decreased or increased the MMP levels compared to those with XJCRTSZW treatment, respectively (Fig. 6a). Transmission electron microscopy showed that there was an increase in mitophagy after EV and XJCRTSZW treatment compared to the TP group. XJCRTSZW treatment combined with inhibition or overexpression of PINK1 reduced or enhanced the mitophagy numbers compared to those in the TP+XJCRTSZW group (Fig. 6b). EV and XJCRTSZW treatments enhanced the TP-induced diminishment of the LC3-II/LC3-I expression, and significantly reduced the TP-induced elevation of the p62 expression.

Besides, the relative protein expression of p62 was prominently increased or decreased by XJCRTSZW treatment combined with inhibition or overexpression of PINK1 compared to the TP + XJCRTSZW group. Furthermore, EV and XJCRTSZW treatment further notably enhanced the TP-induced elevation of PINK1 and Parkin protein levels, while XJCRTSZW treatment combined with overexpression of PINK1 further increased PINK1 levels (Figs. 6c and 6d).

In brief, we concluded that XJCRTSZW ameliorates TP-induced oxidative stress injury by facilitating mitophagy via the *in-vitro* PINK1/Parkin signaling pathway.

## Discussion

In the present study, adult female SD rats and human ovarian GC lines were treated with TP and then with XJCRTSZW. We found that XJCRTSZW ameliorated TP-induced oxidative stress through mitophagy both *in vivo* and *in vitro*. Mechanically, mitophagy mediated by the PINK1/Parkin signaling pathway was involved in this process both *in vivo* and *in vitro*.

TP has multiple pharmacological effects, including anti-inflammatory, anti-arteriosclerosis, and anti-tumor, and has been widely used in clinics. However, a growing body of evidence has shown that TP can induce severe reproductive toxicity both in animals and humans.

In the present study, severe reproductive toxicity was also observed after TP treatment, as evidenced by seriously pathological symptoms, the diminishment of primary follicles and secondary follicles, and an increase in *in-vivo* atretic follicles, which was consistent with the previous study^13^. Moreover, XJCRTSZW treatment notably antagonized the TP-induced pathological symptoms and changes of primary follicles, secondary follicles, and atretic follicles numbers.

The hypothalamus-pituitary-ovary axis (HPOA) modulates ovarian function, the female menstrual cycle, and fertility function via the feedback regulation mechanism of hormones^14^. Therefore, dysregulation of HPOA can result in female endocrine dysfunction and infertility^15^.

Our results revealed that XJCRTSZW treatment significantly elevated the TP-induced reduction of E2 and AMH, but decreased the TP-induced enhancement of FSH and LH. E2 is the most abundant and active estrogen in women. It is secreted by follicular GCs and regulated by LH and FSH, which can promote the development of various organs of the reproductive system, facilitate endometrial hyperplasia and shedding, and maintain female secondary sexual characteristics^16^. AMH is secreted by GCs of small ovarian follicles, and its levels are not affected by other exogenous hormone drugs or pregnancy. Thus, it is a reliable indicator for evaluating ovarian reserve and premature ovarian failure^17^.

Both LH and FSH are secreted by the basophils of the anterior pituitary. Therein, FSH can facilitate the proliferation and differentiation of GCs in the follicle, promote the maturation of the follicles, and make the ovaries grow. LH and FSH have a synergistic effect to promote the discharge of mature eggs, so that the ruptured follicles form a corpus luteum to secrete estrogen and P^18^. Therefore, the levels of E2, AMH, LH, and FSH can directly reflect the functional status of the ovaries. Taken together, we demonstrated that XJCRTSZW can ameliorate TP-induced reproductive toxicity via HPOA.

Plenty of studies have reported that TP can induce oxidative stress both *in vivo* and *in vitro* 9. Similarly, our data showed that XJCRTSZW treatment significantly elevated the TP-induced reduction of SOD, but decreased the TP-induced enhancement of MDA and ROS levels both *in vivo* and *in vitro*. SOD is an antioxidant enzyme that specifically scavenges oxygen free radicals in the body and plays an important role in the body’s oxidation and antioxidant balance. SOD can dysregulate superoxide anion free radicals to produce hydrogen peroxide, which is further converted to water by GSH-PX in the body^19^. MDA is a degradation product of lipid peroxides, reflecting the peroxidation degree of body fat. Thus, SOD activity and MDA content can indicate the degree of oxidative stress.

Moreover, *Cistanches Herba* 20, *Cuscutae Semen* 21, *Rubi Fructus* 22, *Rehmanniae Radix Praeparata* 23, *Spatholobi Caulis* 24, and *Lycopi Herba* 25, the core composition of XJCRTSZW, have been shown to have antioxidant effects in various diseases. Elevated levels of ROS can alter the balance between pro-oxidants and antioxidants. Therefore, appropriate ROS is necessary for the normal progression of cells^26^. Excessive ROS induction is unsuitable for normal female physiological reactions, which result in a series of reproductive diseases, such as PCOS, endometriosis, and infertility^26^^,^ 27. Therefore, these results indicated that XJCRTSZW can ameliorate TP-induced oxidative stress.

Our results also showed that XJCRTSZW treatment observably reduced the TP-induced elevation of apoptosis rate both *in vivo* and *in vitro*. Except for apoptosis, autophagy can be also activated primarily in GCs^9^. Moreover, emerging evidence exhibited that autophagic death is activated under oxidative stress conditions without inducing apoptosis in several mammalian cells^28^^,^ 29.

Our results showed that XJCRTSZW treatment markedly increased the number of mitophagy, and XJCRTSZW treatment also prominently enhanced the TP-induced reduction of ATP and MMP levels. Moreover, XJCRTSZW treatment significantly elevated the levels of LC3-II/LC3-I, PINK1, and Parkin, and reduced the expression of p62 compared with TP treatment both *in vivo* and *in vitro*.

Mitophagy is triggered when the mitochondria are damaged, which can protect cells from different cytotoxic stimuli through the elimination of dysfunctional mitochondria^30^. As a mitochondrial serine/threonine protein kinase, PINK1 plays a core role in modulating mitochondrial dynamics, trafficking, and quality control^31^^,^ 32. Parkin, an E3 ubiquitin ligase, has been also identified as a pivotal regulator of mitochondrial quality control. Moreover, Parkin can promote the PINK1-directed autophagic clearance of depolarized mitochondria^33^^,^ 34. PINK1 tends to be steady on damaged mitochondria to activate Parkin because the reduction of MMP prevents proteasomal degradation of PINK1.

Furthermore, in the present study, XJCRTSZW treatment combined with inhibition of PINK1 notably increased the apoptosis rate and the levels of ROS, MDA and p62. Conversely, it led to a reduction in the levels of SOD, MMP, LCII/LCI, PINK1, and Parkin. On the contrary, XJCRTSZW treatment combined with overexpression of PINK1 markedly reversed these changes. In addition, previous studies have shown that superfluous mitophagy can remove plentiful mitochondria, thereby causing apoptosis^35^^–^ 37. Also, mitophagy can be activated by oxidative stress^38^. Therefore, we concluded that XJCRTSZW facilitates mitophagy via the PINK1/Parkin signaling pathway.

## Conclusion

Our results showed that XJCRTSZW can ameliorate TP-induced oxidative stress through mitophagy both *in vivo* and *in vitro*. Mechanistically, the involvement of the PINK1/Parkin signaling pathway in mediating mitophagy is evident.

## Data Availability

The datasets used or analyzed during the current study are available from the corresponding author upon reasonable request.
